# Hypothermia: a cool therapeutic approach for AD-HSP?

**Published:** 2014-08

Hypothermia is induced in a wide range of clinical settings to prevent tissue injury; for example, lowering the body temperature after cardiac arrest or stroke can minimise consequent neurological damage. A new study by Nina Tang Sherwood and co-authors sought to investigate the effects of cooling in a *Drosophila* model of autosomal-dominant hereditary spastic paraplegia (AD-HSP), following the fortuitous observation that cold temperatures modify symptoms of the disease in this model. AD-HSP is a neurodegenerative disorder characterised by progressive loss of mobility. Mutations in the gene encoding spastin, a microtubule-severing protein, are the most common cause of AD-HSP. Sherwood and colleagues applied cold treatment, during discrete developmental stages, to spastin-null animals and flies expressing mutant versions of the human gene. Cooling improved mobility, lifespan and synapse morphology in mutant flies, particularly when applied during a spastin-dependent developmental stage. Intriguingly, the authors provide evidence that cooling can also rescue mobility and synaptic defects that are attributable to mutations in genes that are not linked with spastin function. These findings indicate that cooling could represent a viable neuroprotective therapy for AD-HSP and potentially other neurodegenerative disorders. Further investigations using fly models could provide insight into the mechanisms involved in hypothermia-associated neuroprotection. **Page 1005**



